# Adherence and the Moral Construction of the Self: A Narrative Analysis of Anticoagulant Medication

**DOI:** 10.1177/1049732320951772

**Published:** 2020-08-28

**Authors:** Meredith K. D. Hawking, John Robson, Stephanie J. C. Taylor, Deborah Swinglehurst

**Affiliations:** 1 Queen Mary University of London, London, United Kingdom

**Keywords:** England, United Kingdom, narrative methods, discursive, moral, medicine-taking, BNIM, atrial fibrillation, anticoagulation, qualitative

## Abstract

In this article, we examine illness narratives to illuminate the discursive work that patients undertake to construct themselves as “good” and adherent. Biographical narrative interviews were undertaken with 17 patients receiving anticoagulation for stroke prevention in atrial fibrillation, from five English hospitals (May 2016–June 2017). Through pluralistic narrative analysis, we highlight the discursive tensions narrators face when sharing accounts of their medicine-taking. They undertake challenging linguistic and performative work to reconcile apparently paradoxical positions. We show how the adherent patient is co-constructed through dialogue at the intersection of discourses including authority of doctors, personal responsibility for health, scarcity of resources, and deservingness. We conclude that the notion of medication adherence places a hidden moral and discursive burden of treatment on patients which they must negotiate when invited into conversations about their medications. This discursive work reveals, constitutes, and upholds medicine-taking as a profoundly moral practice.

## Introduction

Pharmaceuticals play an increasing role in the daily life of patients, driven by the combination of prescribing for chronic disease management and prescribing to reduce risk of disease (and associated health care costs), and partly by a burgeoning market in medications for long-term conditions (Bell & Figert, 2012; Hardon & Sanabria, 2017). There is pressure on clinicians to “optimize” medicines and various cognitive models (Chapman et al., 2015; Clifford et al., 2008; Cooper et al., 1982; Munro et al., 2007) have emerged which guide prescribing clinicians to assess patients’ attitudes and beliefs toward medication and identify barriers to medication use (Murdoch et al., 2014; NICE, 2015). In recent years, professional discourse has shifted away from paternalistic notions of compliance (Parsons, 1951) toward “medication adherence” (World Health Organization [WHO], 2003), which foregrounds the *agreement* between patient and provider (WHO, 2003). Patients are framed as active partners in their care, and the emphasis shifts toward facilitating patient choice (including treatment refusal), and reducing blame (NICE, 2009). In practice, however, medication adherence is often indistinct from compliance (Trnka, 2014), and critics have long argued for alternative conceptions of medicine-taking that more accurately account for patient agency, social context, meaning making, and experience (Britten, 1996; Chamberlain et al., 2011; Conrad, 1985; Dew et al., 2014; Donovan & Blake, 1992; Drabble et al., 2019; Huyard et al., 2017, 2019; Lumme-Sandt et al., 2000; McCoy, 2009; Murdoch et al., 2014; Polak, 2017; Polak & Green, 2020; Pound et al., 2005; Shoemaker & Ramalho de Oliveira, 2008; Webster et al., 2009; Werremeyer et al., 2017). For example, “medicines resistance” captures the creative, hidden work that patients undertake to minimize, modify, and resist medication intake (Chamberlain et al., 2011; Murdoch et al., 2013; Pound et al., 2005). Britten (1996) categorized medication accounts as orthodox—justified by medical rationale—or unorthodox—self-legitimated, with medicines seen as unnatural, ineffective, or potentially harmful. More recent work focuses on the routinization of medicine-taking and the invisible work of negotiating adherence in competing social spaces (Huyard et al., 2017, 2019; McCoy, 2009; Senteio & Veinot, 2014). May’s (2014) Burden of Treatment theory identifies adherence work as requiring significant effort from patients and their social networks. It requires the necessary social skills to secure cooperation, show structural resilience in the face of adversity, and draw on social capital through access to information and material resources. However, previous research has not yet delineated the particular ways in which medication adherence as a wider discourse shapes patients’ accounts about medicine-taking. In this article, we conceptualize talk about medicines as part of the work, or burden (May et al., 2014) that patients with long-term conditions must manage in their everyday interactions.

Medication adherence is ubiquitous in contemporary health care as a guiding principle for treatment delivery (NICE, 2009) and as a benchmark for success and comparison between medical providers (Seabury et al., 2019). In recent years, consultancy firms have developed algorithms for medical data sets that predict future nonadherent behavior and prompt clinicians to adapt their prescribing (FICO, 2019). Interest is also turning to the potential impact of financial incentives for adherence to drugs (DeFulio & Silverman, 2012; Noordraven et al., 2017; Priebe et al., 2013; Volpp et al., 2008). Western media promote the message that “misuse” of medicine has a negative impact on the health system and on patients (Ledwith, 2013). The *New York Times* describes nonadherence as an “out of control epidemic” that is “100% preventable by the very individuals it afflicts” (Brody, 2017). As health systems foreground the importance of individuals taking responsibility for their own health, medication adherence becomes a governing technique signifying the worth and identity of the patient (Dew et al., 2015). Nonadherence is simultaneously depicted as a patient-related problem to be solved by the health sector, and as a personal failing. This invites moral assessments from others regarding how patients use their medicines.

Morality is concerned with guiding what is deemed right or wrong in particular contexts. A moral “assemblage” can be conceptualized as comprising circulating public discourses on morality, institutional discourses on morality, and the personal, embodied dispositions that allow for good or acceptable ways of living in the world (Zigon, 2010). Moral dimensions of medicine-taking are shaped by social, political, and historical contexts. The discourse of medication adherence speaks to power differentials between medical practitioner and patient (Murdoch et al., 2014) and intersects with discourses of personal responsibility and blaming of individuals for ill health (Hill, 2010; Lebesco, 2010), particularly in reference to “intentional” nonadherence. Alongside notions of self-management and patient activation, medicine adherence allows for quantifiable categorizations of “appropriate” patient behavior. Preventive medications, predominantly used by older people, can be used to exert control over the aging body, in line with neoliberal rationales of positive aging and good citizenship (Laliberte Rudman, 2015; Lawless et al., 2017). In the United Kingdom, awareness of resource scarcity within the publicly funded National Health Service (NHS) leads to judgments about the deservingness of other service users (Goode et al., 2004; Griffiths & Hughes, 1994). In this context, nonadherence becomes understood as wasting resources and encouraging avoidable disease, with associated higher societal and individual costs. Indeed, it has been argued that once a patient accepts treatment, they have an obligation to adhere (Glannon & Ross, 2002; Resnik, 2005), else they can be considered “lazy,” “crazy,” “difficult,” and “defiant” by doctors (Resnik, 2005). Accounting for medication adherence becomes a disciplining technique of the self, aligned with shifts toward healthism and responsibilization in neoliberal societies (Crawford, 1980; Kang & Stenfors-Hayes, 2015; Lupton, 1993; Rose, 1999).

Going beyond biomedical conceptions of “social desirability bias” in adherence studies, social scientists have highlighted the complex moral conditions and repertoires that govern medication-related talk and usage (Dew et al., 2015; Lumme-Sandt et al., 2000; McMullen & Herman, 2009; Murdoch et al., 2013; Polak, 2017). Lumme-Sandt et al. (2000) found older patients presented themselves as morally responsible by explaining and minimizing their medication use and comparing it with others who took more medicines. Similarly, medication talk by nonadherent patients is structured in ways to avoid moral judgment (McMullen & Herman, 2009; Murdoch et al., 2013). Medication adherence remains a powerful, morally laden discourse that is (re)produced in talk about medicine-taking. In this article, we delineate how medication adherence manifests and is (re)produced in patient narratives about medicine-taking, as they work to construct and manage adherent selves despite conflicting positions and tensions. We draw on biographical narratives concerning the use of long-term anticoagulants among patients with atrial fibrillation (AF).

AF is a cardiac arrhythmia which increases the risk of ischemic stroke. It affects 1.5% to 2% of the population, with prevalence increasing with age (Camm et al., 2012; Zoni-Berisso et al., 2014). Anticoagulant medication for AF prevents around 16,100 strokes a year in the United Kingdom (Kerr, 2014), but patients taking anticoagulants are more likely to develop bleeding complications. For many years, warfarin, which requires regular blood tests for INR (international normalized ratio) monitoring, has been the U.K. anticoagulant of choice. Since 2012, direct oral anticoagulants (DOACs)—apixaban, rivaroxaban, dabigatran, and edoxaban—have been available. DOACs require yearly renal function tests (not INR monitoring), and have fewer drug and food interactions. In this study, we focus on the narratives of patients with AF within this shifting context of anticoagulant prescribing.

Narrative inquiry is concerned with life experiences narrated by those who live them (Chase, 2013). Narratives can be imagined as emerging from a process of temporal ordering and “sense-making” at a given moment in time (Sandelowski, 1991). They are everywhere, in many guises: spoken narratives range from lengthy biographic life stories (Wengraf, 2001) to “small” stories embedded in everyday talk (Bamberg & Georgakopoulou, 2008). To narrate one’s life is to account for one’s self and emerge as a person through the telling (Riessman, 2003). This is a creative process, shaped in part by dominant narratives that are available in the social world. Narratives are not objective accounts, but occasions for creating, revealing, re-producing, and re-shaping situated subjectivities of the self through interaction. The role of the audience, or listener, in the co-construction of narratives is central. Goffman (1959) developed a dramaturgical metaphor of narrators “performing” desirable, persuasive selves for their audience—real or imagined—shaping the content and form of talk with the listener always in mind (Salmon & Riessman, 2013).

The (co-)construction of a narrative is a moral performance. Bury (2001) defines moral narratives as those that introduce an evaluative dimension into the links between the personal and the social—shame, blame, or religiosity, for example. Narrating illness experience to an audience can lead the teller to examine their life in moral terms (Hydén, 1997). Several narrative studies have explored this moral work (Catedral, 2017; Drabble et al., 2019; Holdsworth & Robinson, 2008; Knutsen et al., 2011; Murdoch et al., 2013; Riessman, 2003; Ryan et al., 2010; Silva, 2013; Williams, 2002). Silva (2013) showed how working-class adults narratively framed emotional self-management and overcoming life’s obstacles as a matter of individual choice, resulting in harsh moral critiques against others who did not achieve self-transformation. Riessman (2003) analyzed how a positive masculine identity was narratively achieved despite health, employment, and marriage “failures.” Ryan et al. (2010) outlined the moral work undertaken by breastfeeding mothers to maintain or repair their subjective position in relation to motherhood. In this article, we extend this body of work with a focus on how people shape their narratives about medicines taking through implicit reference to a moral imperative of becoming and being a “good” and adherent patient. Through revealing this hidden effort, we create new avenues for conceptualizing and understanding the “work” of taking and talking about medicines in research and clinical spaces.

## The Research

We conducted a narrative interview study with 17 patients with AF, recruited from outpatient anticoagulant clinics across five hospitals in England. The clinics were selected to reflect diversity in terms of demography, geography, and type of anticoagulant prescribed. Patients above 18 years old who had been diagnosed with AF and who were prescribed an oral anticoagulant (OAC)—either warfarin or DOAC—for the prevention of stroke were opportunistically given an invitation pack with information about the study when they attended their appointment. Interested patients could then be contacted by telephone or in writing to discuss the study and ask questions. Patients were informed both verbally and via the patient information sheet that participation was voluntary and would not affect their care in any way. Patients then responded by posting a signed consent form. We contacted patients to verbally confirm eligibility and consent, and then to arrange an interview. We sampled purposively, aiming for maximum diversity of anticoagulant-related “experiences,” including exposure (type of anticoagulant, whether they had switched medications), gender, age, ethnicity, and experience of previous cardiovascular events. Recruitment stopped when we had a sufficient range of “anticoagulant experiences” and iterative analysis suggested we had reached “information power” (Malterud et al., 2016). Patients with cognitive disabilities, those who were housebound, or undergoing palliative care were not included as recruitment took place in outpatient clinics. All interviews were conducted in English.

We conducted interviews between May 2016 and June 2017 using the Biographic-Narrative-Interpretive Method (BNIM; Wengraf, 2001). Ten patients were interviewed at their home, and seven in a university office. We piloted the interview schedule with a patient from the project advisory panel who had AF and experience of taking both warfarin and DOACs. The advisory panel also discussed ethical issues, reviewed patient-facing materials, and provided feedback on project design and delivery. Interviews were structured in three parts, beginning with a *single question used to induce narrative* (SQUIN; Wengraf, 2001; Sub-session 1, SS1). The SQUIN invited participants to tell their AF story, beginning where they wanted and including events and experiences that were important to them. We did not interrupt and took brief notes. After a short break, selected cue phrases (actual words spoken by the participant) were used to elicit further, more detailed narratives (Sub-session 2, SS2). Cue phrases were used as prompts in the order in which the participant had introduced them in SS1. Finally, we used a short semi-structured interview schedule (Supplementary File), based on the research questions, to enquire about topics not yet covered in SS1 or SS2 (Sub-session 3, SS3). Formal audio recordings of the entire interview lasted between 32 and 138 minutes, consent for which was provided in writing (consent form) and verbally. We spent additional time with participants during the comfort breaks and after the formal interview which provided opportunity for informal talk and observations which were gathered in a field note journal in addition to a reflexive research journal. These notes provided additional contextual information during analysis. Interviews were transcribed verbatim and anonymized. Participants received £25 vouchers for their time.

## Data Analysis

We adopted a pluralistic narrative analytical approach that combined structural (Labov & Waletzky, 1997), thematic (Braun & Clarke, 2006), and metaphorical (Lakoff & Johnson, 2003) lenses. We began by checking the transcripts for accuracy—we carefully listened to the audio and checked each transcript twice, while cross-checking and adding important contextual information and nonverbal cues from field notes. Familiarization was reached through repeated reading of the transcripts. We then employed structural, thematic, and metaphorical techniques of analysis as different “ways of seeing” the data. Initially, we undertook these approaches sequentially; as our analysis progressed and we gained increasing familiarity with the data, we were able to integrate these approaches, moving back and forth between different analytic lenses guided by our iterative analysis and emerging findings.

We classified segments of narrative from SS1 according to Labov and Waletsky’s (1997) six categories of clauses (Supplementary File 2). We then plotted the main events in a timeline. We noted how narrators shifted positions through attention to evaluative clauses. We annotated the transcripts with codes by hand, and continued coding, using NVivo software for data management (QSR International Pty Ltd, 2012) in six stages (Braun & Clarke, 2006; Supplementary File 2). We identified emerging metaphors line by line and grouped them conceptually according to vehicle (subject) (Supplementary File 2) for comparison within and across transcripts. Metaphors related to body parts (e.g., heart, brain, blood, circulation system), clinical processes (e.g., diagnosis and health care interactions), drugs, and other health-related experiences. Alongside the cross-case processes outlined above, we maintained an analytic commitment to the “whole story” by collating case findings, reflexive, and analytic notes within individual summary documents. To further refine the analysis and increase rigor, the research team discussed findings from each analytical stage, generating new and alternative interpretations and acting as “critical friends.” We adapted Wengraf’s (2001) model of “panel work” through organizing and conducting three analysis panels, each lasting 2 to 3 hours and focusing on a single transcript. Using “reflective teams” (Jones, 2003) or panels can strengthen research findings (Corbally, 2014) and help push the research process along by bringing multiple interpretations to the fore. These interpretations were discussed, pulled apart, and put back together through interactions with others, enriching our “sociological imagination,” and increasing interpretive depth (Corbally, 2014; Meares, 2010) and trustworthiness through peer examination (Phoenix et al., 2016). Each panel consisted of four to six academic researchers (all experienced qualitative researchers, doctoral level or above, specializing in disciplinary areas including anthropology, sociology, medicine, public health, linguistics) to interpret three diverse transcripts from the interviews.

Ethical approval was granted by the North West—Liverpool Central Research Ethics Committee, REC Number: 15/NW/0884. Sponsorship approval was provided by the Joint Research Management Office on behalf of Queen Mary University of London. Local research and development approvals were sought and granted by the Hospital Trust research team for each of the participant identification centers in the study.

## Findings

We interviewed nine women and eight men. In addition, one participant’s daughter took part directly, and another participant’s husband was present but did not contribute verbally. Eight patients had taken warfarin only, seven had switched from warfarin to a DOAC, and two had taken a DOAC only. Participants ranged from those in their mid-40s to those in their 80s (two were 45–59; five were 60–69; eight were 70–79; two were 80–85). Participants self-identified their ethnicity: 12 White British, one Black African, one Black Caribbean, one Chinese, one Indian, and one White Irish. Thirteen had been diagnosed with AF over a year previously. Seven had experienced cardiovascular events: (stroke, myocardial infarction, major bleed). Nine participants were prescribed at least three medications in addition to OACs, thus experiencing polypharmacy. Biographical narratives involve emotional and mental work and have potential for silencing particular voices—some may have trouble in creating a coherent event-focused account (Wengraf, 2001) or may lack the discursive resources with which to make sense of their experience and render it tellable to others. Without these resources one is at risk of suffering what Fricker (2007) refers to as hermeneutical injustice. One participant, who stated she was “beginning to lose the plot a bit” struggled with the SQUIN, but participated more comfortably in SS3.

In this section, we will outline: how participants constructed adherent selves—morally and discursively—through references to gratefulness, expert doctors, and “good” medicine practices and through comparison of themselves with nonadherent others. We show how they managed the conflicting moral positions of being adherent to medicines and also “not somebody who takes medicines” and how they maintained an adherent self while also reporting nonadherent practices. We also show how they dealt with tensions between being adherent to medicines and also taking pleasure in consuming alcohol and foods which they knew “interfered” with their prescribed regimens.

## The Adherent Self—“I Do Remember to Take My Medication Good”

Narratives about medicine-taking aligned with wider discourses of being a good patient through references to being adherent. Adherence was constructed as a virtuous act, while being nonadherent was undesirable. Participants invested much discursive effort to construct adherent selves. One strategy was through references to unquestioning acceptance of medical advice through trusting “expert” doctors and expressing gratefulness for good fortune in having being “given” medicine. This has been identified elsewhere as a “patient repertoire,” characterized by expressions of contentment with medications (Moen et al., 2009) and references to the authority of doctors (Lumme-Sandt et al., 2000), aligning with traditional notions of ideal patient “sick-role” behavior (Parsons, 1951) and altruistic doctors (Glannon & Ross, 2002). Here, a participant, who worked in health care and had experienced a life-threatening episode prior to her diagnosis, positioned herself as accepting of the consultant’s direction and fortunate to have been prescribed a “very high dose” of warfarin:

I was happy to take a tablet. Yeah, when they said, OK, we’ll put you, the consultant came to me and said put you on warfarin straight, put you on warfarin. And I said, OK, thank you. And I started on a very high dose of warfarin. I was pleased to have that. (Female participant)

Her job within a hospital may have exposed her to discourses of medication adherence and sensitized her to successful ways of positioning herself as adherent. In another respondent’s narrative, being adherent to warfarin was also tied to being grateful for medicines, but in this case, she constructed her good fortune by contrast to others who would not have had access to them in the past. The respondent hinted that she tried not to “feel sorry for herself,” pointing to a reluctance to take her medicine but immediately followed this up by clarifying that she was “very fortunate that we live in, at this time, because there’s always medication and it couldn’t be a better time.”

In talk about “good” medicine-taking, most participants described how it had become a “habit” and part of their daily routine. One participant described it as follows: “I take three in the morning and two at night and I never forget. Well I say never but I never forget.” His use of “never” here as an extreme case formulation convinces the listener of his correct behavior (Pomerantz, 1986). Similarly, another respondent repeated that he forgets to take his medication “very rarely, very rarely.” To avoid forgetting or missing a dose, participants referred to using visual and automated reminders, such as using a “special basket.” One participant was prompted by her television programs, and another kept his medicines with the breakfast items. They also described strategies to ensure they could take their medicine while avoiding conflicts with social expectations. For example, one person stated, “I’m always prepared, I always take medication with me.” Like others, his repeated use of the maximum proportion “always” here substantiates his claim that this is the acceptable “right” thing to do (Pomerantz, 1986), solidifying his moral position.

## Adherent Self Versus “People Who Are Given Medication and Will Not Do It”

Another discursive strategy for articulating the adherent self was by contrasting the “good” self with nonadherent others, who were constructed as being ungrateful, wasteful, or acting childishly or inappropriately. One participant, whose mother had also taken warfarin, recalled that her mother was always “playing” with her tablets so they were “everywhere” on the floor and tucked down the side of the armchair and she would “sit with a nail file and file them down!” leading her to describe her mother as “hard work.” Her mother’s actions provided contrast to her own more responsible orientation toward her medicines. Another narrator stated that she was “good” with medicines through references to having faith in doctors and nurses, and not understanding those who are prescribed medication and do not take it:

I’m very good with medication as is my husband. I just wonder what these people, what world they live in when they do these things, but I suppose they must do it. I’ve still got faith in a lot, in doctors and nurses. . . . As I say, I can’t understand people who are given medication and will not do it. I know you often, you see it don’t you sometimes, people are found or they happened and they find all this medication that they’ve, has never taken, all stacked up in the cupboard. (Female participant)

In this passage, she likens the prescription of medication to a gift with the term “given,” and she engages in a clear example of “othering” emphasized rhetorically through use of a three-part list (Jefferson, 1990): the actions of “these people”; from “what world”; who do “these things”—and who by implication are not very good with medication. Her metaphoric hint to their living in a different “world” accentuates the gulf between her own virtuous practices (and those of her husband) and “these people’s” morally dubious practices. She then goes on to make plain that being “good with medication” means taking it and not having it “stacked in the cupboard.” During the rest of her narrative, this interviewee referred repeatedly to being “of the old school” and “doing what she’s told,” to show her acceptance of medical advice without question. Similarly, a different respondent recounted how some friends take unprescribed diabetes medication from America, to which she was very opposed, and repeated “I don’t believe in taking these things. I don’t believe in that.” Her story gave her space to construct her adherent self by highlighting the stark contrast between herself and her friends. Narratives about the self can be constructed in terms of sameness and difference to help explain who the narrator is, and who they are not (Byrne, 2003). For these patients, this difference could be characterized as those who are “good” with medicines, and those who are not.

Comparisons with others in terms of gratefulness and adherence often invoked complementary discourses around wastefulness of medical resources. By indicating the medication “all stacked up in the cupboard,” one could assert that “these people” are inappropriately storing and wasting medicines and hint to their eventual discovery as a matter of shame. Gratefulness for medicines links to wider discourses of scarcity in the NHS, which provokes moral condemnation when people are perceived as resource-wasters and time-wasters (Cromme et al., 2016; Goode et al., 2004). For example, one person recalled watching a documentary about the ambulance service (an “eye opener”), explaining “when I watched it, one woman and one man: ‘I’m hungry. That’s why I need an ambulance to take me to the hospital to get fed.’ I thought, yeah all right mate.” Talk about medicines intersected with moral assessments about appropriate use of medical services, drawing on and reproducing discourses of deservingness for “underfunded” health care. Indeed, the desire to avoid being perceived as an irresponsible consumer of health care was so strong for one patient that she avoided calling an ambulance while she was experiencing a myocardial infarction because she was “worried about wasting someone’s time.”

## Adherent Self Versus “I’m Not Somebody Who Takes Tablets”

Dependence on medications is commonly viewed negatively and patients are often reluctant to take medicines, or minimize their use (Benson & Britten, 2002; Britten, 1994; Lumme-Sandt et al., 2000; Polak, 2017; Pound et al., 2005). It is important to avoid being labeled as an “abuser” of prescription drugs by peers or professionals (Butler & Sheridan, 2010). The virtuous position of being adherent to long-term anticoagulants conflicted with a general reluctance toward taking medicines or *being seen as* someone who takes medicines. As one female patient explained, “I don’t like taking medication. But I know now I have to.” This presented a tension which narrators managed in their talk, often offering apparently contradictory accounts, or adopting multiple positions simultaneously. One narrator, for instance, described himself as a “habitual creature . . . so I take God knows what” including fish oils and vitamin tablets. Following this, however, he said that he was “not somebody who takes tablets.” He explained this further:

I’m grateful that I’m taking it [edoxaban] because I feel safer. I think that’s the thing, but whether it actually does anything for, you know, I had a bad knee and I go to the doctor and they say, “do you want painkillers?,” I’m like “I don’t want painkillers, I’ll have smarties I’ll just cope with it.” So I’m not somebody who takes tablets, but I feel protected if you know what I mean, that’s what I feel. (Male participant)

One way participants managed these conflicting positions was by reference to different properties or categories of medicines. Stronger adherence rules applied to anticoagulant medications than painkillers, for example. This was because anticoagulants were constructed as important or “special” medicines, needed to keep one alive. When a male patient told his doctor he wanted to stop taking warfarin, the doctor said she would “get the ambulance ready” which convinced him that discontinuing warfarin would represent a real and immediate threat to his health, and so he continued taking it. His account of the interaction displays his resistance to taking medication and simultaneously legitimizes his continued use of warfarin by removing the notion that he has a choice, due to the necessity of warfarin for survival.

The moral judgments and penalties relating to nonadherence for anticoagulants were greater than those meted out for other medications. For instance, another respondent, who had undergone a successful catheter ablation (a surgical procedure to stop abnormal electrical activity in the heart tissue) and had previously asked her doctor when she might be able to “come off” warfarin, described how she planned to discontinue her tablets for blood pressure and cholesterol prior to a clinic appointment, but would not consider doing this for warfarin:

Participant: I have the feeling that if you ask, “can I come off it?” They might be reluctant, so if I just do it for four weeks beforehand and they might be able to see.

Interviewer: So what, if you come off the warfarin or?

Participant: Not the warfarin, no, I’ll keep, I must keep the warfarin. . . . Oh I don’t mess with that [warfarin], yeah, no, no, no.

Interviewer: What’s the difference between the warfarin for you and Ramipril?

Participant: Because the others can be sorted out by diet and lifestyle, versus having a stroke or a heart attack, well pushing up daisies and all that sort of thing. No, no, no, I hope I, I think I understand that very well. (Female participant)

As shown by this passage, she would never consider “messing” with her warfarin (she emphasizes this twice in this extract with the persuasive repetition of “no, no, no”)—positioning it as fundamentally different to her other medications. Here, medication nonadherence was more or less acceptable depending on the type of medicine, and whether the effect it had was thought to be achievable through “diet and lifestyle” changes. She balances tensions between two social discourses—that of the virtue of medication adherence, and of discourses that promote the maintenance of a healthy, slim body through physical effort and self-control rather than resorting to or depending on pills (Broom & Whittaker, 2004; Crawshaw, 2007; Gonsalves et al., 2016; Lupton & Chapman, 1995). People relying on medicines instead of doing the work to keep themselves healthy could be considered lazy and immoral, lacking in self-control (Lebesco, 2010). Avoiding these labels is important to be considered a worthy patient (Higashi et al., 2013); however, it presented tensions for participants. One participant, who had experienced a myocardial infarction emphasized his “really, really fit” health status and contrasted this with other people who he considered “massive, absolutely massive.” Another admitted that she was previously overweight but then clarified “I wasn’t huge, I wasn’t blubber waddling around, I can’t bear to see it.” She then went on “you don’t even think you’re fat or you’re overweight until suddenly you realize you are. So that going on and on at someone just wouldn’t work, you just alienate people.” In the same moment, she resists, and reproduces, the stigmatizing discourse about overweight bodies.

## Adherent Self Versus “I’m Not So Great With Timings”

Some participants worked narratively to overcome a paradox—that of maintaining a “good,” adherent self, while also reporting missing, skipping, or stopping their medicines. The medication adherence discourse shaped how they told their stories so that they could avoid inviting negative judgment from the researcher.

When patients described nonadherent practices, they did so in ways that convinced the interviewer that they knew they had behaved inappropriately. These stories sometimes took the structure of a confession, where nonadherence was justified by excusable reasons ranging from “stupid mistakes” to lapses of memory accompanied by feelings of guilt, or references to ways the narrator could “improve” themselves next time. One patient, for instance, recounted how she realized “in a panic” that she had forgotten her tablet and organized a reminder to avoid reoccurrence. Socially acceptable reasons for missed doses included occasional forgetting, being ill, being fearful of side effects, and changes to daily routines. Bury (2001) highlights the ubiquitous nature of the confessional in moral narratives. Take this quote in which a participant followed each admission that she sometimes takes her anticoagulant medicine a bit later than she “should” by a self-chastisement, and said that she “gets annoyed with herself”:

So I get up in the morning, I have a cup of coffee and then I have my, take my tablets. But then on the Monday and Thursday when it’s my days off I’m not so great with timings. And I do know I should sort that out. Perhaps keep my dossette box upstairs or in the en suite or, I do need to get better. Or even with refilling the dossette box. If I’m just remembering to take them out of the drawer it’s not, probably, it’s probably a couple of hours later than it should be. Which I should know better really. (Female participant)

In this example her repeated comments about knowing and being better can be seen as a defense strategy against negative judgment. She later minimizes the occurrence of nonadherent medicine practices, referring to them as “the odd times” and links them to changes in her everyday routine or the location of her medicines. These shifts in inhabited spaces and everyday scheduling are known to shape the strategies that patients must use to adhere effectively (Huyard et al., 2019). Throughout her narrative she made many comments expressing concerns about what other people might think about her actions—including being considered a “fraud” on online forums. Like being a fraud, being nonadherent was an undesirable position, which she undertook discursive work to avoid.

Narrators did not only refer to missed or forgotten doses, but also to dosage alterations. Here an interviewee admitted:

I have to be honest, sometimes I balanced it [dosage] myself by thinking, no, that doesn’t sit right with me, that you’re going to put me from 1.5 warfarin to suddenly 3.5. That was scary. So I’d think, no, I’m going to do 2.5, and I’m going to do that for two days, and then, and that’s what I found myself doing. (Female participant)

Her confession works to absolve her of blame, by owning up to her behavior “I have to be honest”; through reference to fear of taking too much “scary” medication; and through hints at the unintentional nature of her actions—“that’s what I found myself doing.” In another example, a male participant talked about nearly taking too much medication, and described this as a mistake:

Well, if you make a mistake and overdo it I think it can cause internal bleeding, I nearly did the other day actually [laughs]. But, I’d already taken the tablets first thing and then I was filling, I’ve got one of these dispensers, the seven day boxes, and I was getting out a couple of 5mg ones to put in and I put them in my mouth. And I thought, no, that’s not right. I’d have taken 15mg that day, if I’d not stopped, it’s so stupid. (Male participant)

Here he avoids potential judgment by avoiding blame for his accidental actions, using humor as a defensive strategy (Drabble et al., 2019) and also through rectifying his “near miss” at the time. He draws on the common understanding that mistakes happen occasionally and can be rectified. As these examples show, virtuous adherent selves were maintained with discursive practices that helped avoid negative judgment and allowed the informants to save face (Goffman, 1959; Murdoch et al., 2013).

## Adherent Self Versus “I’ve Got to Enjoy Myself”

In the context of AF and anticoagulants, being a “good,” adherent patient didn’t only involve taking medicines. For those who took warfarin, rules governed consumption of certain foods and drinks especially alcohol, green leafy vegetables, certain fruits, and other medicines and supplements. In the following passage, one narrator explained how he regulates his alcohol intake to avoid affecting his INR levels or AF symptoms:

If I have a birthday and somebody buys me a bottle of gin, [slaps the back of his hand] naughty, naughty. But what I’ve done, I’ve just had a birthday and what I’ve done is if I have a gin and tonic now I make sure that I have two capfuls, I undo the top just to, otherwise what I used to do, [gestures free pouring] brrr, so you didn’t know how much I was putting in. [Laughter] So I have a couple of capfuls and it’s fine. (Male participant)

Although his INR measurements had been “up and down a bit,” he maintains his virtuous status in several ways. By slapping his hand and repeating the term “naughty,” he invokes a chastised child, absolving himself of blame for indulging in alcohol. Discursive positioning as children has been documented among patients with diabetes to excuse deviance from recommended lifestyle changes (Broom & Whittaker, 2004). In this quote, he also describes changing his behavior to be more careful about the amount of gin he drinks and further assures the interviewer he is “not a drunken” and only has “one or two,” to avoid negative associations with alcohol overconsumption or dependence and loss of control (Butler & Sheridan, 2010). He appeals for understanding and exemption from this stigmatizing identity by linking drinking with special occasions, in this case his birthday. Similarly, another interviewee described eating green vegetables “once in a while” and one mentioned she only had “Asti” at Christmas, unlike “silly youngsters.” Digressions from approved diets were constructed as treats, to be indulged in small amounts, every now and again. Indulging in prohibited treats can be used as a way to sweeten the moment (Warin et al., 2015) for those living with chronic health conditions or in adverse circumstances, or as a way of carving out time for self-care in busy schedules of work and care roles (Graham, 1987).

Following the rules was not easy and required ongoing self-control and work. As one respondent pointed out, to avoid things interacting with warfarin, “you have to study.” Another person recounted this conversation with a nurse:

She [nurse] said, “Do you treat yourself, [patient name]?” And I said, “Once in a lifetime.” So she said, “Do you eat cakes?” I said, “No,” I said, “but the only thing,” I said, “I do sometimes like a jam doughnut.” She said, “[patient name], treat yourself to a jam doughnut now and again.” She said, “Don’t,” she said, “you’re one of the good ones,” she said, “Because you can, you’re able to control it yourself.” And I know that there’s people in this world even younger than I am who have everything done for them. They put, have somebody round saying, stop and take this, time you took this. But I control it. (Male participant)

In this excerpt, he legitimizes his occasional “treats” in multiple ways. Alongside his reference to having a doughnut “once in a lifetime,” the nurse’s reported speech characterizes him as “one of the good ones” due to his self-control and mastery. Comparing himself with younger people who are less independent situates his bodily control and resistance to temptation as exceptionally good compared with other people. By pointing out the constant work and vigilance that is required, he justifies an occasional release in the form of a sugary doughnut. He therefore manages tensions between being seen as someone who adheres to the rules, and also legitimately deserves an occasional reward for these efforts.

## Discussion

In this article, we have shown how circulating discourses around medication adherence shape and are shaped by moral talk about medicines. We have illustrated the ways in which a “good” adherent self was constructed and maintained in moral narratives in the face of tensions that arise from concerns about being a pill-taker, and have shown how language offers flexible resources that patients can draw on when reporting skipping, missing or stopping medicines, or indulging in drinking and eating prohibited foods. The ethical self must be managed in line with other intersecting discourses around the authority of doctors, responsibility for one’s health, the scarcity of biomedical resources, and deservingness. Talk in illness narratives arising in research settings is constrained by and reinforces these moralizing discourses, and participants must undertake challenging linguistic and performative work to reconcile paradoxical positions.

The interviewer was complicit in the co-construction of the “good” adherent patient self, which is shaped by rules of social desirability. Her previous expertise in public health and research on medicine-taking among other patient groups (Hawking et al., 2017; McNulty et al., 2015) meant she was aware of how common nonadherence is, and previous failures by the research community to meaningfully improve medication adherence prompted her to take a more critical perspective when exploring medicine-taking and how this evidence is constructed. Medical roles and gender have been shown to influence qualitative research, the data one collects, and the research context (Nelson, 2019; Richards & Emslie, 2000). In our case, female participants mostly chose to be interviewed at home, whereas all but one male opted to visit the university. While the interviewer communicated to patients that she was not a doctor, and interviews were held in nonclinical settings, she was tied to the clinic, in part because NHS ethical approval requires patient materials to have clinical references, logos, and biomedical terminology. Most participants had, unprompted, prepared a pile of medical letters, records, and medicines prior to the interview. Some structured their SS1 narratives as clinical histories (which they were well rehearsed in), with little evaluative talk and modest narrative detail. When nonmedical details were discussed in the initial narratives, some participants would qualify them with comments such as “but you don’t want to know about that.” Biomedical discourses therefore undoubtedly shaped—and may well have constrained—patients’ accounts of their adherent selves, the data we were able to collect, and may even have pushed them to work harder than usual to resolve moral and positional tensions in their accounts. In response to these challenges, the interviewer worked reflexively and flexibly, adapting by maintaining an open and nonjudgmental position, building rapport, and reassuring patients that there were no right or wrong answers, and increased prompting for more detail in SS2 with “felt” or “emotional” cue phrases rather than “clinical detail” cues. This often led to more candid sharing in later stages of the interview.

Using a SQUIN (Wengraf, 2001) reduces the interviewer’s control in the interview, giving the narrator more freedom and space to construct their own accounts compared with alternative interview techniques. This can increase the potential credibility of the data. However, there is potential to end up collecting “thin” data, or for participants to find it difficult to tell their story in an unprompted session, and careful prompting or flexibility in utilization of the method is required to avoid facilitate sharing in this case. We ensured trustworthiness of our analysis through the systematic use of multiple analytic lenses, which allowed for different “ways of seeing” beyond a single analytic approach. Furthermore, the data and interpretive findings were critically examined and developed through panel analyses with experienced qualitative researchers, and the research team acted as a “critical friends” at every analytic stage. Finally, we kept a reflexive journal, notes of which were included in the case summary reports alongside analytic notes and findings.

Our findings may provide some insight into why reported adherence in research studies is highly variable across different medication types and regimen complexity. It may be that the additional work of adhering to more challenging medication regimes, or to “special” medications such as anticoagulants affords an elevated moral position—adherence in the face of particular challenge or burden. This consideration may be particularly pertinent in circumstances where adherence becomes visible through monitoring and measurement, and a potential subject of moral judgment in questionnaires and interviews. As researchers who are interested in medicine-taking, we may reinforce or reproduce these opportunities for ethics to emerge, upholding medicine-taking as a moral practice.

We included patients with a diverse range of experiences and backgrounds from a broad geographic area and differing clinical contexts, to increase the potential transferability of our findings. However, all participants who responded to our invitation self-selected, and would probably be considered adherent by medical professionals, where definitions of “acceptable” adherers include those who take anticoagulants according to prescription 80% of the time, (Mueller et al., 2017), or who self-report that they usually undertake adherent behavior. None of the interviewees reported discontinuing their anticoagulants or changing, skipping, or forgetting doses “often” in their accounts. We were unable to recruit any patients who had independently discontinued their anticoagulant medication, in part a limitation of our approach to recruitment, which took place in medical settings with clinical staff. We reassured patients that taking part would not affect their medical care; however, the findings are therefore most pertinent to patients who—broadly speaking—consider themselves to be adherent medicine-takers. That patients are invested in constructing and maintaining an adherent self in medical research settings provides insight into why those who discontinue or modify their anticoagulant regimes might not have responded. Not responding can be interpreted as a form of resisting judgment, of rejecting a request to account for one’s own actions in line with discourses that blame and moralize decisions about and practices around medicines. Like others (Murdoch et al., 2014), our findings problematize the binary categorization of people as “adherent” and “nonadherent” that is common in biomedical research on medicine-taking. This binary belies the complexity of medicine-taking as a practice and encourages comparisons between patients.

We have shown that in some situations moral discourses may support medicine-taking, but may also prevent disclosure of nonadherent practices. For clinicians, supporting patient medicine-taking is challenging in short clinical appointments, particularly in light of our findings. Medication reviews are key opportunities to support patients—but questions must be nonjudgmental and open to invite talk from the patient agenda and overcome the pressure for patients to perform linguistically as “adherent.” This would contribute to efforts toward patient-centered care and true shared decision-making (Charles et al., 1997; Constand et al., 2014; Mead & Bower, 2000). Clinicians must expect medication practices to change over time, particularly for long-term medications, and see adherence as an ongoing, complex practice that requires regular follow-up rather than a one-off assessment or binary categorization. Medication taking can be supported by addressing concerns and focusing on practical solutions (Chapman et al., 2015; NICE, 2009), but this still relies on patients being able to openly discuss their medicine-taking without judgment. Challenging moralizing discourses with comments such as “many patients struggle with taking their medicines, and this does not mean they have done anything wrong” may also be useful. All this may not be effective, however, if clinicians continue to linguistically reproduce circulating discourses around the authority of doctors and personal responsibility for health, which may also drive the “good” patient narrative.

The normative medication adherence discourse is not neutral; it has consequences which can be unhelpful to patients and practitioners. It takes significant discursive work to construct and maintain an adherent self and benefit from the morally acceptable position it affords in the eyes of others, including researchers and health care professionals. Drawing on May’s (2014) Burden of Treatment Theory, we can interpret this discursive effort—or discursive burden—as an additional facet of the work identified as “social skill” that is required to secure cooperation and collaboration from those in relational networks, including making the most of opportunities to access and utilize health care resources. Patients within these networks are accountable for their behavior and governed by expectations about what is right and good. Notions such as “shared decision making” and “autonomous, empowered patients” (Charles et al., 1997; Constand et al., 2014; Mead & Bower, 2000) may be at risk of buckling under the discursive pressure of medication adherence and the moral imperatives it summons for patients in their encounters with professionals. By pressuring patients toward being “good,” this discursive burden has the potential to close down candid conversations that would actually help support long-term medicine-taking in a patient-centered way. We should be aware of this burden for patients who decide to take medicines and are called upon to discuss their medicines regularly in clinical spaces (and sometimes in research spaces). While the concept of adherence claims to offer patients autonomy in medicine related decisions (WHO, 2003), patients must work hard to avoid moral condemnation when they make decisions which conflict with norms governing “appropriate” illness behaviors. Our findings suggest that clinicians may also have to work hard to achieve a genuine patient-centered consultation in this context, recognizing the discursive burden experienced by patients taking medicines and providing opportunities for additional support. To overcome the pressure to be “good” and adherent, patients may need extra space to express their medicine-related concerns and perceptions without judgment, and clinicians need to create this space.

## Supplemental Material

sj-pdf-1-qhr-10.1177_1049732320951772 – Supplemental material for Adherence and the Moral Construction of the Self: A Narrative Analysis of Anticoagulant Medication Click here for additional data file. Supplemental material, sj-pdf-1-qhr-10.1177_1049732320951772 for Adherence and the Moral Construction of the Self: A Narrative Analysis of Anticoagulant Medication by Meredith K. D. Hawking, John Robson, Stephanie J. C. Taylor and Deborah Swinglehurst in Qualitative Health Research

sj-pdf-2-qhr-10.1177_1049732320951772 – Supplemental material for Adherence and the Moral Construction of the Self: A Narrative Analysis of Anticoagulant Medication Click here for additional data file. Supplemental material, sj-pdf-2-qhr-10.1177_1049732320951772 for Adherence and the Moral Construction of the Self: A Narrative Analysis of Anticoagulant Medication by Meredith K. D. Hawking, John Robson, Stephanie J. C. Taylor and Deborah Swinglehurst in Qualitative Health Research
