# Supplementary material for: Adherence and the Moral Construction of the Self: A Narrative Analysis of Anticoagulant Medication
Source: Qual Health Res. 2020 Aug 28;30(14):2316–30. doi: 10.1177/1049732320951772 (PMC7649927; doi:10.1177/1049732320951772)
Supplement: sj-pdf-1-qhr-10.1177_1049732320951772 – Supplemental material for Adherence and the Moral Construction of the Self: A Narrative Analysis of Anticoagulant Medication [file sj-pdf-1-qhr-10.1177_1049732320951772.pdf]

Supplementary file 1 – Hawking et al. 2020. *Adherence and the moral construction of the self*.

Interview outline

Sub session 1: SQUIN

As you know, I'm researching people's experiences of atrial fibrillation. So can you please tell me your story of atrial fibrillation, from when you first noticed something of relevance up to now, including all the events and experiences that were important for you personally?

I'll listen first. I won't interrupt. Please take your time. I'll just take some notes in case I have any follow up questions after you've finished telling me about it all.

Sub session 2: follow up narratives, based on participants' cue phrases, in the order in which they were told.

Sub session 3: semi-structured questions

Diagnosis

- Can you tell me about when you were first diagnosed with atrial fibrillation?

Perceptions:

- What does AF mean to you?
- Can you describe in your own words what happens to a person when they have atrial fibrillation?
- How does it feel?

Stroke risk:

- Are you aware of any problems that having atrial fibrillation can cause?

OAC Therapy:

- What comes to mind when you think of your [anticoagulant] medication?
- Can you describe for me in your own words how anticoagulation works?
- What are the benefits for you of taking [anticoagulant] medication?
- Are you aware of any risks associated with taking [anticoagulant] medication?
- [For DOAC patients previously on warfarin] How does your [DOAC] medication compare to warfarin?
- Can you walk me through how you manage taking your [anticoagulant] medication?

Monitoring

- How often do you see a health care professional about your [anticoagulant] medication?
- [Warfarin] What effect, if any, does the monitoring have on your day to day life?

Information:

- Is there anything about atrial fibrillation or anticoagulation that you would like to know more about?
- What types of information would be useful?
- How would you like to receive this information?
