# Supplementary material for: Adherence and the Moral Construction of the Self: A Narrative Analysis of Anticoagulant Medication
Source: Qual Health Res. 2020 Aug 28;30(14):2316–30. doi: 10.1177/1049732320951772 (PMC7649927; doi:10.1177/1049732320951772)
Supplement: sj-pdf-2-qhr-10.1177_1049732320951772 – Supplemental material for Adherence and the Moral Construction of the Self: A Narrative Analysis of Anticoagulant Medication [file sj-pdf-2-qhr-10.1177_1049732320951772.pdf]

Analytic concepts

Structural lens: Six categories of clauses
1. Abstract (what the story is about)
2. Orientation (who, when, where, what)
3. Complicating action (what then happened?)
4. Evaluation (so what?)
5. Resolution (how did it end?)
6. Coda (signal that the story is finished)

Thematic lens: Six stages
1. Familiarization through repeated reading and checking
2. Generating initial codes and discussion with second researcher
3. Searching for themes by coding all transcripts and generating initial categories
4. Reviewing themes with other researchers, develop thematic framework and check coherence
5. Defining and naming themes, review and develop findings within each sub theme
6. Finalize themes and findings, discuss summary reports with other researchers

Metaphorical lens: conceptual outline

Metaphor allows for the understanding and experiencing of one thing (x) in terms of another (y), referred to as the vehicle (x) and the target (y). Metaphorical concepts are thus denoted: x is y, e.g. *argument* is *war*. In this example, *arguments* are partially structured, understood and performed in terms of *warfare*.
